# Insight into climate change from the carbon exchange of biocrusts utilizing non-rainfall water

**DOI:** 10.1038/s41598-017-02812-y

**Published:** 2017-05-31

**Authors:** Hailong Ouyang, Chunxiang Hu

**Affiliations:** 1 Key Laboratory of Algal Biology, Institute of Hydrobiology, Chinese Academy of Sciences, Wuhan, 430072 China; 2 University of Chinese Academy of Sciences, Beijing, 100049 China

## Abstract

Biocrusts are model ecosystems of global change studies. However, light and non-rainfall water (NRW) were previously few considered. Different biocrust types further aggravated the inconsistence. So carbon-exchange of biocrusts (cyanobacteria crusts-AC1/AC2; cyanolichen crust-LC1; chlorolichen crust-LC2; moss crust-MC) utilizing NRW at various temperatures and light-intensities were determined under simulated and insitu mesocosm experiments. Carbon input of all biocrusts were negatively correlated with experimental temperature under all light-intensity with saturated water and stronger light with equivalent NRW, but positively correlated with temperature under weak light with equivalent NRW. LCPs and R/Pg of AC1 were lowest, followed in turn by AC2, LC2 and MC. Thus AC1 had most opportunities to use NRW, and 2.5 °C warming did cause significant changes of carbon exchange. Structural equation models further revealed that air-temperature was most important for carbon-exchange of ACs, but equally important as NRW for LC2 and MC; positive influence of warming on carbon-input in ACs was much stronger than the latter. Therefore, temperature effect on biocrust carbon-input depends on both moisture and light. Meanwhile, the role of NRW, transitional states between ACs, and obvious carbon-fixation differences between lichen crusts should be fully considered in the future study of biocrusts responding to climate change.

## Introduction

Arid and semiarid regions account for 41% of global land area, while more than 70% soil surface of these regions is occupied by biocrusts mainly composed by cyanobacteria, fungi, lichen and moss. So biocrusts are the primary biological components of the soil carbon pool of these areas^1^, and play an important role in topsoil stabilization, texture improvement and fertilization enhancement^1–4^. Due to the location of the topmost soil surface, the sensitivity to environment, and their simple composition and easy manipulation, biocrusts are model ecosystems to study both land climate change^5–9^ and ecological restoration of drylands^10, 11^.

There have been many simulation studies on biocrust responses to climate, and the consistent trends include carbon emission increase, cyanobacteria increase, lichens decrease, succession retrogression^9, 12–20^, and nitrogen fixation was negatively affected^21–23^, even the community shift was similar to that of physical disturbance^19^. However, there were quite a few conflicting results. In terms of warming alone, some reflected that the coverage and abundance of lichens or/and mosses was reduced^6, 9, 17–19, 24^. Some reported that the coverage of lichens distinctly decreased but mosses increased slightly^7^, or community composition, the function and soil chemistry were only slightly affected or not affected^13, 15, 16, 21^, or lichens were less affected^19^. As to the effects of altered precipitation, the composition and function of biocrusts were not significantly influenced, or only negatively affected in certain areas or seasons^5, 7, 9, 13, 17, 21^, or completely contradictory in the same geographic profile^25, 26^. The above inconsistent results were partially ascribed to the diversities between regions, day and night, and seasons^5, 9, 18, 27, 28^, but we thought that the genetic differences from evolutionary positions^19^, and the complex interactions between temperature, water^6, 9^ and light intensity were both the fundamental and long standing questions. So the intrinsic characteristics of dominant communities in biocrusts should be specially considered, and the pattern of their carbon exchange responding to crucial environmental factors must be clarified.

Of all the environmental factors, water is most important. Without water other factors will not exert effect. Once moistened, the wetness degree, temperature and light intensity would all significantly affect biocrusts^6, 7, 12, 29–31^. However, the natural biocrusts are inactive in more than 90% time due to desiccation^32^. So the present detections of CO_2_ exchange in biocrusts were performed either under indoor simulation experiments that artificially added water^33–38^, or after rainfall^39^. Additionally, a striking advantage of biocrusts was their utilization of non-rainfall water (NRW), which occurred ca. 270 nights every year^40, 41^ and accounted for a high proportion of the total annual precipitation^42^, even was the only water source^43–46^. Some also suggested that NRW was of great importance in carbon balance of biocrusts^9^. However, by now there are few studies on the NRW utilization by biocrusts.

Light, as the necessary condition for the carbon fixation of cryptogams, is the driving energy of the earth’s temperature change. Either seasonal or diurnal carbon exchange at global scale influenced by temperature is actually driven by solar irradiation. Since photosynthesis of most plants mainly occurs in daytime with abundant sunlight, previous studies paid more attention to the effects of temperature and water rather than light^5, 18, 47^, thus the influence of light on carbon exchange of biocrusts is also poorly known.

NRW accumulation occurs in the period from nightfall to early morning, with variable temperature, light intensity and NRW amount, and this is an ideal natural opportunity to study the interactions between biocrusts carbon exchange and water, temperature and light. Therefore in this study, we selected 4 types of biocrusts that are highly dominated by variant cryptogams and possess different carbon assimilation mechanisms, namely 2 cyanobacteria crusts (AC1 and AC2), 1 lichen crust with cyanobacteria as photobiont (LC1), the other with green algae as photobiont (LC2) and 1 moss crust (MC). In order to obtain intrinsic potentiality and adaptation patterns of biocrustal carbon exchange responding to temperature, light and water, various light intensities and temperature conditions that were similar to the nights with abundant NRW, as well as two moisture gradients of saturated water (SW) and equivalent NRW (E-NRW) were firstly designed. The respiration rate (R), net photosynthesis rate (Pn), gross photosynthesis (Pg), and light compensation points (LCPs) of different biocrusts were measured, and the pattern of CO_2_ exchange were synchronously analyzed by multivariate nonlinear regression. The ratios of respiration to gross photosynthesis (R/Pg), which could reflect the biological differences of various biocrust types utilizing water, were also explored. Then, by *in situ* mesocosm experiments, CO_2_ exchanges of all chosen biocrusts were analyzed in NRW abundant seasons, the crucial factors and the patterns of CO_2_ exchange were determined by structural equation models, and the influencing degrees were quantifiably perceived by path coefficients. Finally, the community changes of biocrusts under the background of global climate change were predicted, and the suggestions for subsequent relative studies were proposed.

## Results and Discussion

### Dark respiration (R), net photosynthesis (Pn), light compensation points (LCPs), gross photosynthesis (Pg), and R/Pg under saturated water

**Figure 1:**
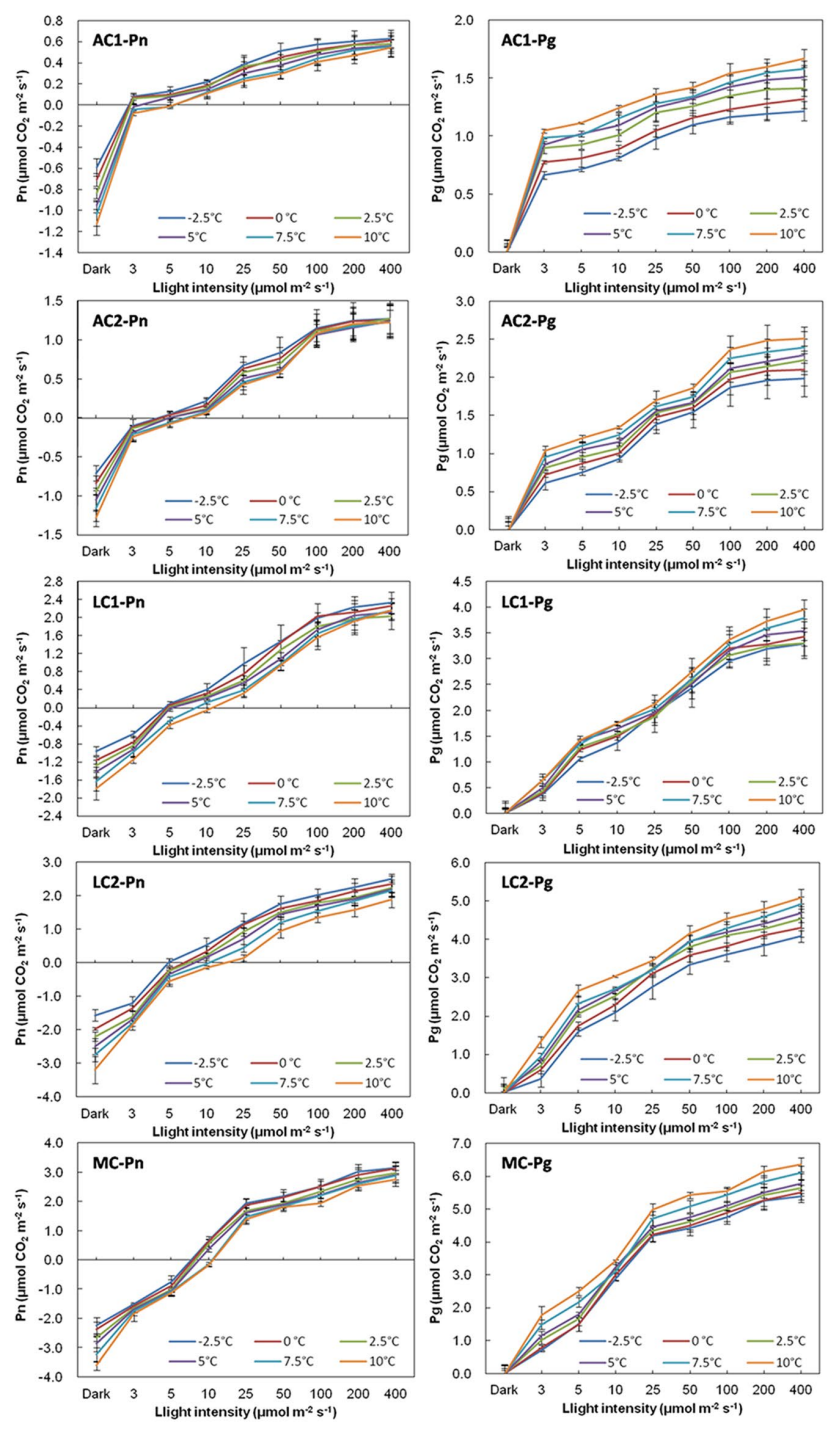
Dark respiration, Pn and Pg of biocrusts determined under different light and temperature conditions when water is saturated. Carbon exchange rate under dark condition represented respiration. Pg is calculated from R and Pn at the same temperature. (n = 3~5).

**Figure 2:**
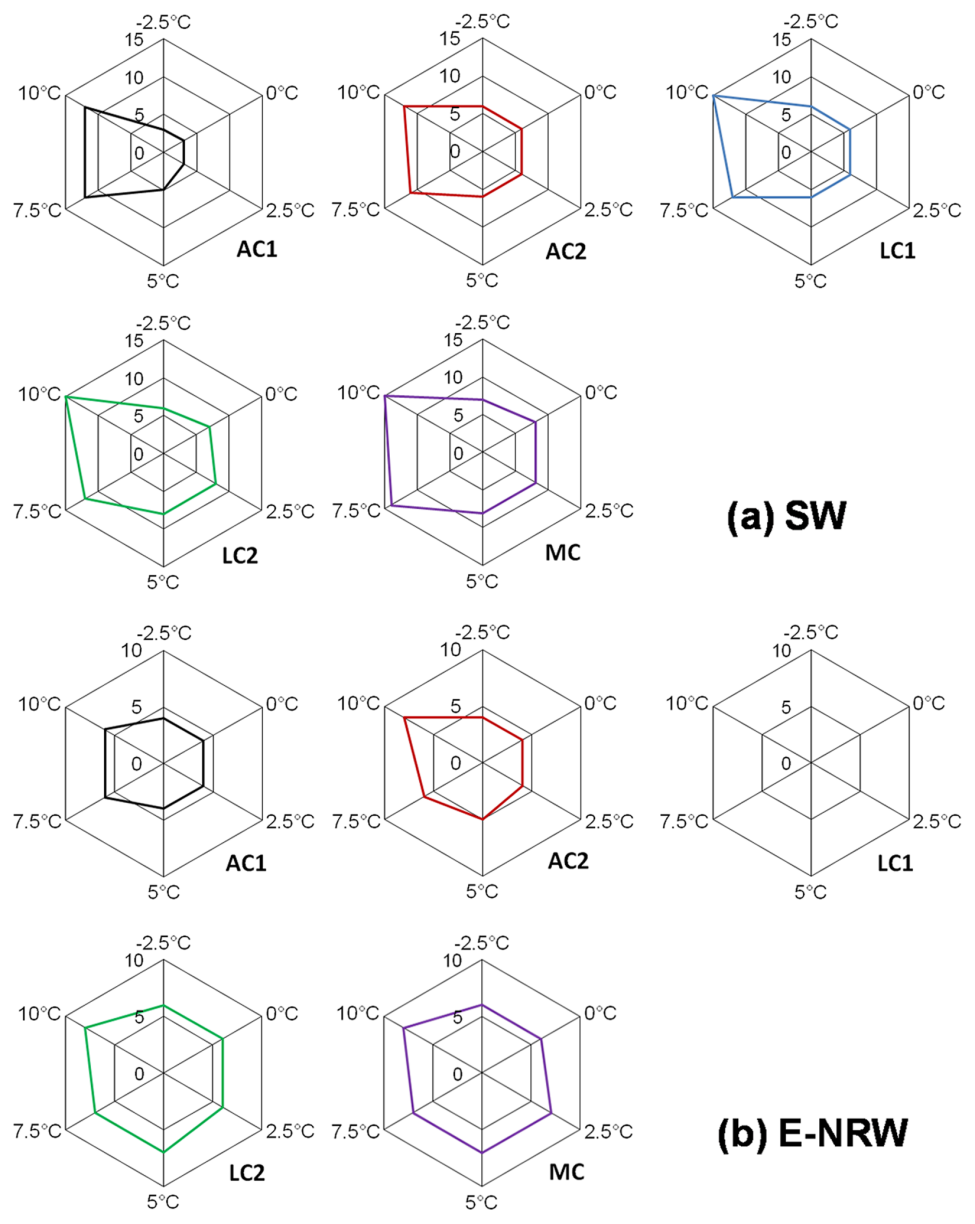
Light compensation points of biocrusts under two water conditions (SW and E-NRW) and different temperature gradients.

R of all biocrusts increased as temperature increased (Fig. 1), but at the same temperature, the order from high to low was: MC > LC2 > LC1 > AC2 > AC1. Pn obviously increased with light intensity, but decreased with rising temperature for the same biocrusts, though no significant difference over different temperatures. LCPs also varied with temperature, specifically, the lower the temperature, the lower the LCP. At the same temperature, the LCP of AC1 was the lowest, and in turn followed by AC2, LC1, LC2, and MC (Fig. 2a). The pattern of Pg was similar to Pn, namely increased with light intensity and temperature, but the increasing amount was highest in ACs, followed by LCs, and MC was the lowest. At the same temperature and light intensity, the maximum Pg was in the order: MC > LC2 > LC1 > AC2 > AC1. And the difference of 2.5 °C in ACs resulted in quite different Pg.

**Figure 3:**
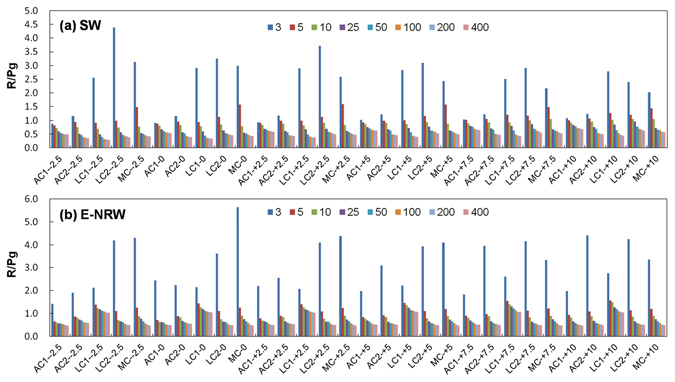
The ratios of dark respiration to gross photosynthesis (R/Pg) for biocrusts at different range of light intensity (3~400 μmol m^−2^ s^−1^) and temperature gradients (−2.5~10 °C) under SW and E-NRW.

For R/Pg (Fig. 3a), it exponentially declined with light intensity, but as temperature increased, the differences among biocrusts gradually decreased and became more similar, especially when light intensity was higher than 25 μmol m^−2^ s^−1^. At 3 μmol m^−2^ s^−1^, R/Pg of AC1 was lowest, followed by AC2, then LCs and MC. At 5 μmol m^−2^ s^−1^ of light intensiy, R/Pg of two ACs were still lowest, that of MC was highest; but under −2.5~5 °C, the order of two lichen crusts was LC1 < LC2, under 7.5~10 °C, LC2 < LC1. Under 10 μmol m^−2^ s^−1^ light, R/Pg of LC2 was highest from −2.5~7.5 °C, LC1 was second-highest from 2.5~7.5 °C, MC was second-highest at −2.5 °C. At 10 °C, the sequence was LC1 > LC2 > MC.

### R, Pn, LCPs, Pg and R/Pg under E-NRW

**Figure 4:**
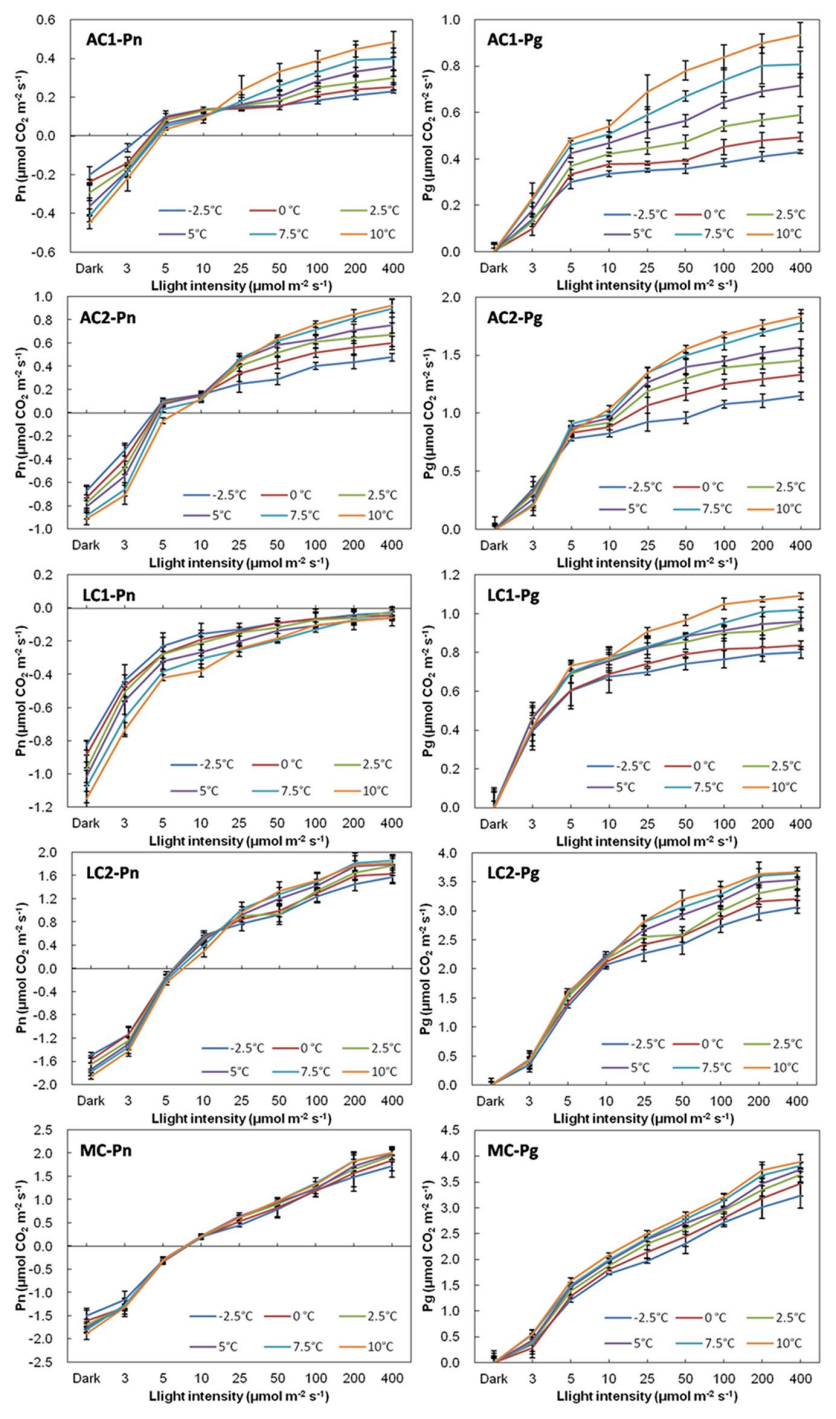
Dark respiration, Pn and Pg of biocrusts determined under different light and temperature conditions when water content is equivalent to NRW. Carbon exchange rate under dark condition represented respiration. Pg is calculated from R and Pn at the same temperature. (n = 3~5).

R of all biocrusts under E-NRW also increased with temperature (Fig. 4), the sequence from high to low was MC > LC2 > LC1 > AC2 > AC1. For Pn and LCPs, both increased with temperature, but Pn (except LC1 was never detected) had no significant differences (P > 0.05) between temperature gradient of 2.5 °C. At the same temperature, LCP in AC1 was lowest, in turn followed by AC2, LC2 and MC (Fig. 2b). Under light intensity lower than LCPs, the time window of positive Pn and the increasing amplitude with decreasing temperature manifested in the order: AC1 > AC2 > LC2 (LC2 slightly above MC). But the order reversed under higher light intensity, the increasing amplitude with rising temperature was in the order of AC1 > AC2 > LC2 ≈ MC. The pattern of Pg was similar to that of SW except for LC1 (Fig. 4), Pg also increased with light intensity and temperature, and the increasing amplitude was also highest in ACs, and then in turn was LCs and MC (Fig. 4a). The magnitude under the same temperature and light intensity from highest to the lowest was MC, LC2, AC2, and AC1. When light intensities were higher than 25 μmol m^−2^ s^−1^, the warming of 2.5 °C in ACs also resulted in quite different Pg.

The R/Pg decreased with the increase of light intensity. Along with the increase of temperature, the differences in R/Pg values among various biocrusts decreased gradually and approached each other under light higher than 25 μmol m^−2^ s^−1^. But at 3 μmol m^−2^ s^−1^, the R/Pg of AC1 was lowest at −2.5~10 °C, and that of AC2 only next to AC1 at −2.5~0 °C. From −2.5~5 °C, R/Pg of LC2 and MC were higher and MC > LC2. The gaps between AC2 and LC2 and MC decreased at 5 °C. At 10 °C, R/Pg of AC2 was highest. Under 5 μmol m^−2^ s^−1^, the R/Pg values were in the order of LC1 > MC > LC2 > AC2 > AC1, and LC1 > MC > AC2 > LC2 > AC1 under 10 μmol m^−2^ s^−1^.

### Comparisons of R, Pn, LCPs, Pg, and R/Pg under SW and E-NRW

The largest difference was that Pn was not detected positive in LC1 under E-NRW (Figs 1 and 4). Small precipitation events are very important in arid and semiarid areas^48^, but the responses of different biocrusts were differently^49–51^. Based on our data, the different responses were mainly determined by their community traits because the low R/Pg meant approaching carbon balance. During NRW accumulation period, AC1 had strongest competitive advantage due to lowest R/Pg under weak light of all temperatures (highest Pn and lowest R). AC2 had advantage because its R/Pg at −2.5~0 °C was second only to that of AC1. Though LC2 could utilize vaporous water to activate photosynthesis and had relatively high assimilation efficiency^52^, its R/Pg at −2.5~5 °C was lower than that of MC. So its competition power just followed ACs. MC well adapt to water-deficit environments due to effectively allocation water^53, 54^, but was less competitive than LC2 due to the highest R/Pg (−2.5~5 °C) and relatively stronger depend on rainfall^50^. For LC1, although it possesses high resistance to light intensity^55^ and well adaption to a wide range of temperature (2~40 °C), but it always was at a loss status under NRW, so we thought it could not use NRW, because the maximum NRW (0.17 mm) could not meet its the minimum requirement (0.21 mm)^52^. This further confirmed that its growth might depend on high photosynthetic efficiency under high temperature and high light after rainfall^52^. Additionally, although MC also needed more water to activate photosynthesis, its higher roughness, larger contact area with atmosphere, and better cooling effect at night^56^ made it acquire the required NRW amount. Except the above cryptogams, dominant heterotrophic organisms might also had different or special traits, and further studies are needed.

Then under E-NRW, except for LC1, the relationships between temperature and Pn showed the opposite tendency before and after 10 μmol m^−2^ s^−1^. Namely, Pn decreased with temperature when light intensity was lower than 10 μmol m^−2^ s^−1^, but increased with temperature when light intensity was higher than 25 μmol m^−2^ s^−1^, while Pn under SW always decreased with the rising temperature. At the night with limited light, Pn is negatively related to temperature under both SW and E-NRW with light lower than 10 μmol m^−2^ s^−1^, which is similar to the pattern of vegetation growth responding to autumnal nocturnal temperature in arid areas of the north hemisphere and spring nighttime of the south hemisphere^28^. Under E-NRW with light intensity more than or equal to 25 μmol m^−2^ s^−1^, Pn is positively correlated with temperature, which also resembled the pattern of vegetation responding to vernal and aestival daytime temperature in arid areas of both north and south hemisphere^28^. This indicated that the relationships between Pn and light intensity and temperature were also regulated by soil moisture conditions^57^. The pattern of Pn was different in diverse moisture ranges due to the adjustment of soil humidity^49^. The negative correlation between Pn and temperature was mainly because the assimilation intensity of autotrophs was stronger than the dissimilation intensity of heterotrophs. The dark respiration of ACs under SW was not much higher than that under equivalent NRW, and the dark respiration of ACs did not increase with water content^47^. The positive correlation between Pn and temperature (light at least 25 μmol m^−2^ s^−1^) was because the assimilation intensity of autotrophs increased more quickly than the dissimilation intensity of heterotrophs with the rising temperature.

Thirdly, Pn under E-NRW was lower than that under SW for the same temperature and light intensity. We know that the carbon sequestration capacities of different stages of biocrusts are significantly different, and that of MC is the highest, followed in turn by LC and AC; or later stages are higher than earlier stages, light-colored biocrusts are lower than dark-colored ones^6, 34, 39^. We also confirmed these patterns both in Pn and Pg. But we thought this did not only depend on biomass of cryptogams, but also on their genetic characteristics. The RuBisCO activities of cyanobacteria are remarkable lower than that of green algae and mosses. Although the two ACs are both dominated by cyanobacteria, AC2 had a higher carbon fixation rate than AC1 under stronger light, because the abundant *Scytonema* of AC2 has advantages in solar radiation tolerance and nitrogen-fixing^4, 55^.

Fourthly, LCPs were significantly influenced by the water condition (Fig. 2a,b), under E-NRW the LCPs decreased and the values were lower than that of SW, except for AC1 at equal or lower than 2.5 °C. And the larger decreasing amplitude occurred at 7.5~10 °C. Under light condition, the CO_2_ exchange of wet biocrust is actually the result of the balance between the assimilation of autotrophic cryptogams and the dissimilation of heterotrophic microorganisms. Though moisture and temperature are most important environmental factors influencing soil carbon cycle^5, 9, 12, 58^, light intensity is the primary factor in the carbon balance between assimilation and dissimilation. The suitable conditions of assimilation and dissimilation are usually not synchronous, but adaptable plants just need low environmental conditions (low temperature, weak light and low water condition included) to activate assimilation^59, 60^. Our experiments showed that under either SW or E-NRW conditions, the light intensity that AC1 required to activate positive Pn was the lowest, followed by AC2 and LC1, and LC2 and MC needed the highest. This was mainly due to their genetic characteristics of dominant cryptogams. The photosynthetic organisms in the two ACs and LC1 are all cyanobacteria, containing abundant amounts of chlorophyll a and phycobilin, allowing them to capture low light that cannot be utilized by other plants. Cyanobacteria also possess CO_2_-concentrating mechanism (CCM), which allows them to absorb and store CO_2_ in carboxysomes when photosynthetic carbon fixation occurs^61^, and thus reduce CO_2_ release, resulting in lower LCPs.

LCP is relatively constant under a certain temperature and increases with temperature^52^. We found that LCPs under SW and E-NRW behaved very differently. The LCPs under E-NRW almost were all lower than that under SW in all experimental temperatures. The decreasing amplitude in the higher temperature range (5 °C~10 °C) was larger than that under temperature lower than 5 °C. This showed biocrusts could regulate LCPs to gain a maximum photosynthetic carbon fixation rate according to the water conditions. Additionally, biocrusts with lower LCPs were more competitive in utilization NRW. And free cyanobacteria possessed stronger CCM than other autotrophs under high water content^6, 32^, so ACs generally had lower LCPs.

Finally, the amplitude of R increasing with increased temperature under E-NRW was lower than that under SW. Dark respiration is an oxidation-reduction process in which soil communities absorb oxygen and release carbon dioxide in darkness. In biocrusts, it generally increases rapidly with the rising temperature^5, 12^, and the thermosensitivity also increases with soil moisture^12^. Our results of respiration were similar to the above patterns, but different biocrusts had disparate sensitivities to soil water and temperature. Specifically, R of AC1 was much more sensitive to water content than temperature, yet that of AC2 and MC, like Grote’s results^6^, were more sensitive to temperature than water content, while the sensitivities of two LCs to the two factors were similar. These differences were closely related to community composition (both autotrophs and heterotrophs), activity and labile organic matter^62^.

### Relationships between CO_2_ exchange and water content, temperature, light intensity

Based on multivariate nonlinear regression analysis (Table 1), temperature, water content were both significant predictors of R (accounted for 83~98% of the total variance). R was positively related to temperature and water content, but the disparities of the explanatory degrees obviously existed among different biocrusts. For instance, 78% variances of R in AC1 was explained by water content, and temperature accounted for more variance (59%) of R in AC2. The predictive power of water content and temperature approached for two LCs, and 69% variance of R in MC was explained by temperature.

**Table 1:** Multiple regression analyses of dark respiration (R) in response to water content (W) and temperature (T), and photosynthesis in response to temperature and light intensity (L).

		L	W *R* ^*2*^	P	T *R* ^*2*^	P	Total *R* ^*2*^	Predicted model
AC1	R	0	0.7819	<0.0001	0.1931	<0.0001	0.9750	−0.161 − 0.887W − 0.032T
AC2	R	0	0.3202	<0.0001	0.5853	<0.0001	0.9055	−0.66 − 0.185W − 0.032T
LC1	R	0	0.4441	<0.0001	0.4693	<0.0001	0.9134	−0.789 − 0.195W − 0.046T
LC2	R	0	0.4290	0.0011	0.3982	0.0014	0.8276	−1.331 − 2.98W − 0.078T
MC	R	0	0.1587	0.0138	0.6880	<0.0001	0.8467	−2.205 − 0.075W − 0.076T
***SW***		**L**	**T** ***R*** ^***2***^	**P**	**L** ***R*** ^***2***^	**P**	**Total** ***R*** ^***2***^	**Predicted model**
AC1	Pn	3–400	0.0231	0.1140	0.5212	<0.0001	0.5444	−0.107 − 0.015T + 0.05$\sqrt{{\rm{L}}}$
	Pg		0.0721	0.0053	0.4941	<0.0001	0.5663	0.607 + 0.028T + 0.027$\sqrt{{\rm{L}}}$
AC2	Pn		0.0083	0.1921	0.7501	<0.0001	0.7583	−0.226 − 0.015T + 0.099$\sqrt{{\rm{L}}}$
	Pg		0.0308	0.0129	0.7330	<0.0001	0.7637	0.605 + 0.03T + 0.099$\sqrt{{\rm{L}}}$
LC1	Pn		0.0178	0.0319	0.7956	<0.0001	0.8134	−0.449 − 0.038T + 0.175$\sqrt{{\rm{L}}}$
	Pg		0.0072	0.1716	0.8031	<0.0001	0.8103	0.676 + 0.024T + 0.175$\sqrt{{\rm{L}}}$
LC2	Pn		0.0177	0.0917	0.6776	<0.0001	0.6952	−0.787 − 0.049T + 0.208$\sqrt{{\rm{L}}}$
	Pg		0.0380	0.0141	0.6628	<0.0001	0.7008	1.133 + 0.073T + 0.208$\sqrt{{\rm{L}}}$
MC	Pn		0.0099	0.1897	0.7039	<0.0001	0.7138	−1.009 − 0.047T + 0.27$\sqrt{{\rm{L}}}$
	Pg		0.0183	0.0758	0.6975	<0.0001	0.7158	1.399 + 0.064T + 0.27$\sqrt{{\rm{L}}}$
***E*** **−** ***NRW***		**L**	**T** ***R*** ^***2***^	**P**	**L** ***R*** ^***2***^	**P**	**Total** ***R*** ^***2***^	**Predicted model**
AC1	Pn	≤10	0.0576	0.0032	0.8319	<0.001	0.8896	−0.298 − 0.011T + 0.148$\sqrt{{\rm{L}}}$
		≥25	0.4829	<0.001	0.3916	<0.001	0.8745	0.082 + 0.015T + 0.011$\sqrt{{\rm{L}}}$
	Pg	≤10	0.0564	0.0037	0.8319	<0.001	0.8884	−0.052 + 0.01T + 0.148$\sqrt{{\rm{L}}}$
		≥25	0.8392	<0.001	0.1209	<0.001	0.9601	0.329 + 0.036T + 0.011$\sqrt{{\rm{L}}}$
AC2	Pn	≤10	0.0322	0.0478	0.8157	<0.001	0.8477	−0.778 − 0.017T + 0.315$\sqrt{{\rm{L}}}$
		≥25	0.4825	<0.001	0.3968	<0.001	0.8794	0.25 + 0.028T + 0.02$\sqrt{{\rm{L}}}$
	Pg	≤10	0.0005	0.7669	0.8419	<0.001	0.8427	−0.053 + 0.002T + 0.315$\sqrt{{\rm{L}}}$
		≥25	0.7218	<0.001	0.2088	<0.001	0.9306	0.975 + 0.047T + 0.02$\sqrt{{\rm{L}}}$
LC1	Pn	≤10	0.0844	<0.001	0.8693	<0.001	0.9537	−0.89 − 0.021T + 0.246$\sqrt{{\rm{L}}}$
		≥25	0.1792	<0.001	0.6132	<0.001	0.7925	−0.18 − 0.006T + 0.009$\sqrt{{\rm{L}}}$
	Pg	≤10	0.0054	0.1477	0.9429	<0.001	0.9483	0.002 + 0.005T + 0.246$\sqrt{{\rm{L}}}$
		≥25	0.7028	<0.001	0.2152	<0.001	0.9180	0.712 + 0.02T + 0.009$\sqrt{{\rm{L}}}$
LC2	Pn	≤10	0.0101	0.1143	0.9120	<0.001	0.9221	−1.599 − 0.021T + 0.226L
		≥25	0.0999	<0.001	0.7816	<0.001	0.8815	0.637 + 0.025T + 0.057$\sqrt{{\rm{L}}}$
	Pg	≤10	0.0018	0.4993	0.9194	<0.001	0.9212	−0.029 + 0.008T + 0.226L
		≥25	0.3350	<0.001	0.5762	<0.001	0.9110	2.207 + 0.054T + 0.057$\sqrt{{\rm{L}}}$
MC	Pn	≤10	0.0023	0.473	0.9080	<0.001	0.9103	−1.638 − 0.009T + 0.199L
		≥25	0.0245	0.0037	0.9127	<0.001	0.9372	0.193 + 0.018T + 0.089$\sqrt{{\rm{L}}}$
	Pg	≤10	0.0123	0.1028	0.8993	<0.001	0.9116	−0.037 + 0.02T + 0.199L
		≥25	0.1396	<0.001	0.8034	<0.001	0.9431	1.794 + 0.047T + 0.089$\sqrt{{\rm{L}}}$

SW = Saturated water. E-NRW = Equivalent non-rainfall water. Pn = Net photosynthesis, Pg = Gross photosynthesis. L = Light intensity (μmol m^−2^ s^−1^). Partial and total regression coefficients, and total predicted models for R, Pn and Pg are shown, respectively.

Under the two water conditions and the light intensity ranges, Pn and Pg were both positively related to light intensity. Under all combinations of experimental light intensity and SW, light intensity had more influence on Pn than temperature, and Pn was always negatively related to temperature. However, for Pn under equivalent NRW, light intensity had more predictive power than temperature only under light intensity less than 10 μmol m^−2^ s^−1^, and Pn was negatively related to temperature. But the predictive power of temperature was stronger than light intensity that was equal to or more than 25 μmol m^−2^ s^−1^, so Pn was positively related to temperature at this condition.

Regression models also showed that 54~81% variances of CO_2_ exchange were explained by temperature and light intensity under SW. Light intensity accounted for 52~79% variance of Pn and 49~80% variance of Pg. In general, the models had more predictive power for Pg than for Pn. For CO_2_ exchange under E-NRW, temperature and light intensity accounted for 79~96% of the total variance, and 82~94% of the variances were explained by light intensity at low light (≤10 μmol m^−2^ s^−1^). At higher light intensity (≥25 μmol m^−2^ s^−1^), temperature accounted for 48~84% variance of CO_2_ exchange in the two ACs; but its explanatory powers for the other three types of biocrusts were lower, although the explanations for Pg (14~70%) were still significant (<0.0001).

Other than genetic factors, the light intensity is very important to photosynthetic efficiency. Although AC2 also had carbon-fixation advantage under weak light, it had higher Pn and Pg than AC1 under higher light intensity. LC and MC also required a higher light intensity to release their higher photosynthetic productive potential. But compared to the daytime after raining, biocrusts under NRW were actually in a condition with limited light. Thus Pn and Pg of all biocrusts significantly positively correlated with light intensity, and light intensity explained 82~94% variances of carbon exchange under light lower than 10 μmol m^−2^ s^−1^, while 72~84% variances of Pg were explained by temperature under light of at least 25 μmol m^−2^ s^−1^ only for ACs. Variances of Pg were lower (14~70%) for other crusts, because free cyanobacteria had much more advantages over other cryptogams under weak light, and the determination of Pn needed to consider both respiration and assimilation^6^.

Temperature also significantly influences Pn and Pg, and generally Pn and Pg all increases with temperature below their optimum. However, respiration intensity often also increases with temperature when moisture is abundant^12^. To offset and exceed increased respiration consumption, biocrusts must produce higher Pg under the rising temperature. The questions are that the preference temperatures of assimilation and catabolism are not always the same. The increased temperature and decreased moisture all increase respiration, while carbon sequestration increase only when temperature reduction and rainfall enhancement^49^ were typical daytime pattern except for lichen crusts dominated by *Collema* and cyanobacteria crusts, because they had stronger positive response to rising temperature^35, 52^. This was consistent with that ACs was more sensitive to water than temperature^6^.

It is also worth mentioning that according to the present references, light-colored crusts are often cyanobacteria crusts dominated by *Microcoleus*, and their response patterns are basically the consistent, for example, Pg was positive with temperature and negatively with water content^6^. But dark-colored crusts include both cyanobacteria crusts dominated by *Scytonema*, *Nostoc* and *Microcoleus* ^5, 6, 34^ and lichen crusts dominated by cyanolichen^6, 40, 59, 63^, some even were mixed by cyanobacteria and lichens^16–18, 20^. So the present confusion mainly existed in dark-colored crusts. In our results, the relationships between Pn/Pg and temperature under saturated water and E-NRW with weak light (10 μmol m^−2^ s^−1^) were consistent with the present partial patterns^6, 35, 63^; only under the conditions with at least 25 μmol m^−2^ s^−1^ light and E-NRW, carbon exchange was positively related with temperature, just like light-colored crusts from cool desert. So biocrust carbon input under NRW is always positively correlated with light, while the effect of temperature depends upon both the range of water content and light intensity. Namely, when water is limited and light is relative abundant, the effect of temperature is positive; but when water is enough, it is negative, regardless of light intensity. So it is difficult to judge the effect of temperature on carbon flux just by dark-colored crusts or light-colored crusts^6^.

### Relationships between carbon exchange and NRW, temperature and light intensity in the field

**Figure 5:**
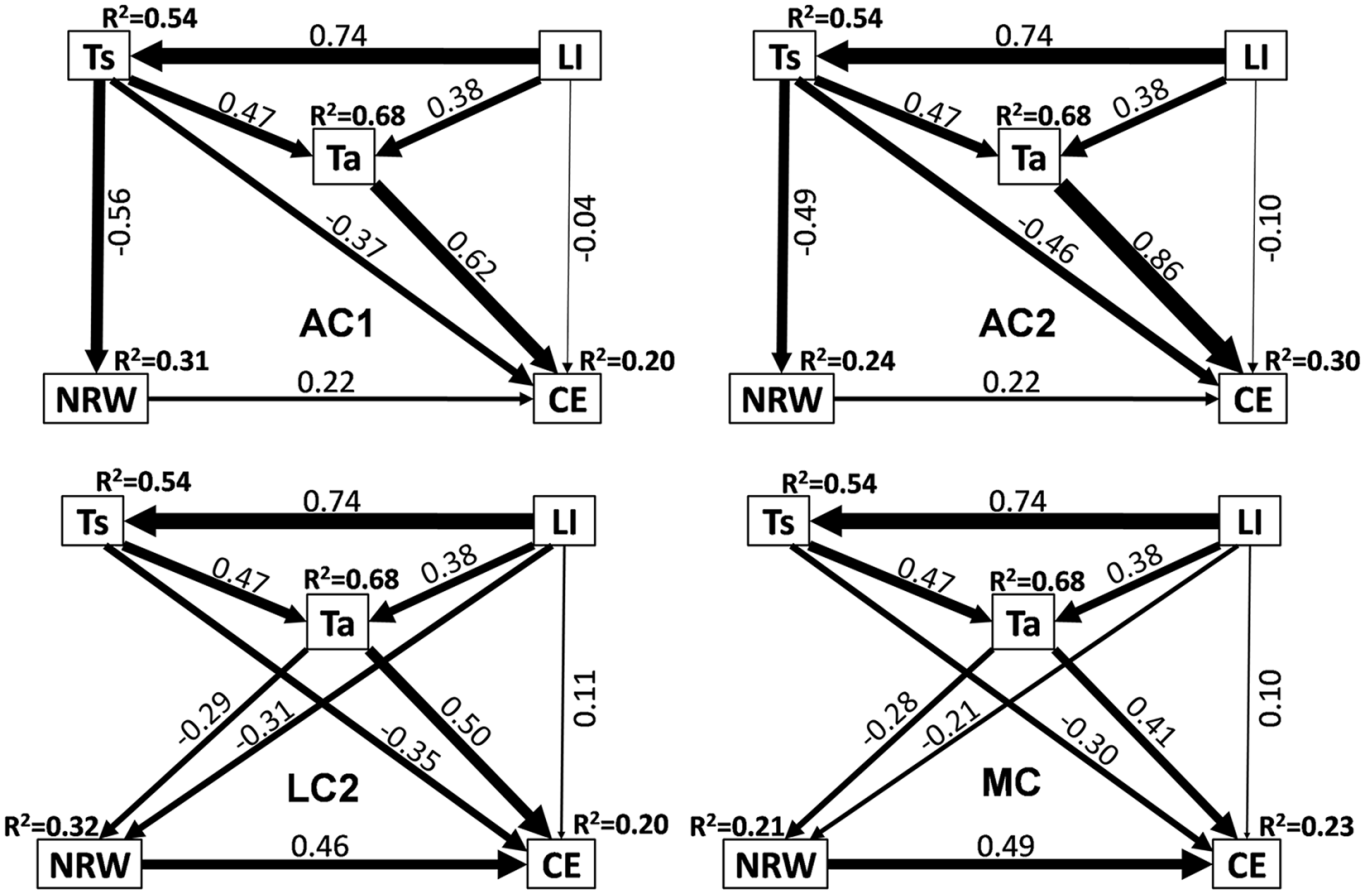
Effects of NRW amount, light intensity (LI) and temperature on carbon exchange (CE) of biocrusts during NRW accumulation period in natural environment. Ta = Temperature of air, Ts = Temperature of soil surface. The width of narrow is in accordance with path coefficient.

Structural equation models (SEM, Fig. 5) based on field data showed that the *R* ^*2*^ values of various biocrusts were at the 0.20~0.30. Variations of surface temperature (Ts), air temperature (Ta), and CO_2_ exchange (CE) were all directly driven by light intensity, and the driving force for Ts was the strongest, followed by Ta, for CE was the weakest. Ts had the strongest effect on NRW in two ACs (Fig. 5a,b). However, in LC2 and MC, light intensity directly affected NRW and also indirectly affected NRW via Ta (Fig. 5c,d). Therefore, CE was influenced by light intensity, Ta, Ts, and NRW together. For the CE of ACs, influence of Ta was the strongest, followed by Ts, then NRW, and that of light intensity was the minimum. For the CE of LC2 and MC, Ta and NRW both had strongest effect, followed by Ts, influence of light intensity was the weakest, but still higher than that of ACs.

### Prediction of climate change

Though the abilities of different biocrust types using NRW are different, the experimental biocrusts except for LC1 all could gain carbon input under NRW, and the carbon exchange patterns are clearly different from that in daytime^6, 7, 28^. Specifically, daytime warming often decreases photosynthesis and increases respiration^18^, while weak light in nighttime with warming increases photosynthesis more and increases respiration less. So the prediction for climate change from carbon balance should include both daytime and nighttime situations.

Although the direct effects of light intensity and temperature on carbon exchange of biocrusts are distinct under different moisture conditions, diurnal and seasonal carbon exchanges are fundamentally driven by solar irradiation. With the aggravation of global warming, more solar shortwave radiation pass through the atmosphere and arrive at the earth, which result in more aggravated changes in air temperature and soil surface temperature, and will further affect the responses of biocrusts to climate parameters. Based on the importance of soil moisture to climate change^50^, together with the negative correlationship between Pn and temperature under nocturnal weak light, we found that warming facilitated the cover of ACs more than that of LC2 and MC; AC2 more than AC1; and LC2 more than MC, and the decreased NRW amount caused by climate change would decrease the cover of LC2 and MC more than ACs. Whereas the coverage of ACs increased more or decreased less than that of lichens and mosses meant that the cover of LC2 and MC would decline on the whole pattern, and this is similar to most of the existing results^9, 12–20, 59^. For LC1, warming alone had little effect, but long-lasting rainfall or frequent NRW without precipitation were detrimental^5, 64^. LC2 has advantage in using vaporous water, so it should only enlarge at the environment with humid or foggy air, and any exsiccation is adverse. For MC, frequent small precipitation events have negative effects but a relative long-time rainfall is advantageous^59^. Overall, warming with frequent NRW (>0.08 mm) is beneficial to the expansion of ACs. So global warming was particularly in favor of enlarging biocrust coverage in Polar Regions^65^ and other arid-cold areas such as the Tibetan Plateau^66^.

In view of biological traits of biocrusts, the increase of coverage of ACs, which is able to tolerate various temperature, drought, salt stress^6, 47, 67^, drought^68, 69^, and disturbance^19^, might explain the most common influences resulting from various and adverse arid environments^7, 13, 19^. We thought the transition states between AC1 and AC2, and LC1 and LC2, and mixed biocrusts reduced the differences of various types^16, 63^, and that was the basic reason of the present confusing results^5, 9, 18, 26, 28, 29^.

## Conclusion

The effect of temperature on biocrust carbon exchange was regulated by water content and light intensity together. Non-rainfall water and light intensity should be considered in the future associated study, and the carbon input in cloudy days after rainfall, at dusk and dawn all should be counted. Additionally, warming under NRW did increase more coverage of cyanobacteria crusts than lichen crusts and moss crusts. But the increased degrees for light-colored (*Microcoleus* dominated) and dark-colored cyanobacteria crusts (with abundant *Scytonema* and *Nostoc*) were distinguishing, the responses of cyanolichen crusts and chlorolichen crusts were even quite different. So the coverage of biocrusts could be applicable for monitoring early alterations caused by climatic changes. And it is greatly urgent to accurately determine biocrust types and community composition, particularly to differentiate the types of lichen crusts and dark-colored cyanobacteria crusts. The comparison of different biocrust types in the same successional stage also should be more accurate. That is, other than the aforementioned NRW and light intensity, the transitional states between ACs, and lichen crusts with obvious carbon-fixation differences all should be fully considered in the future study about biocrusts responding to climate change.

## Material and Methods

### Study areas

The ACs and MC used in our experiments were sampled from our Field Experiment Station (Hobq Desert, 40°21′N, 109°51′E). As a transitional zone of plateau desert and desert steppe, the region is a typical continental monsoon climate with an average elevation of 1040 m and an annual mean temperature of 6.1 °C (the lowest is −34.5 °C and the highest is 40.2 °C). The annual precipitation and potential evapotranspiration respectively is 293 mm and 2400 mm. The average wind velocity is 3.3 m s^−1^ and windy (>5 m s^−1^) day is more than 180 d y^−1^. The soil texture is aeolian sandy soil, and large areas are shifting sand dunes with an average relative height of 5 m. At present, there is an artificial vegetation area of nearly 5000 m^2^ formed by cyanobacterial inoculation, in which there are abundant biocrusts^70^.

### Sampling

Samples of ACs and MC were all collected from Dalate sites, the two LCs from Shapotou Scientific Experimental Station of the Tengger Desert (37°32′N; 105°02′E), where is more drier and the environmental conditions are as Hu *et al*. described^2^. Biocrusts were sampled with a 5 cm-diameter metal ring sampler and transferred to 15 cm-diameter petri dishes at Dalate Site and Shapotou Site in September of 2014 and 2015, after no rainfall events for at least three days. Sampling positions were in open areas at least 0.2 m far away from shrubs and coverage of the same biocrusts type was determined to be basically the same. Biocrusts samples were transported to the laboratory as soon as possible to prepare for experiments. Natural thickness and completeness of biocrusts samples were ensured throughout the process of collection and transportation. The characteristics of the five types of biocrusts are shown in Table 2.

**Table 2:** Characteristics of various biocrusts in different succession stages (mean ± s.e., n = 3).

	AC1	AC2	LC1	LC2	MC
Colour	Grey	Black brown	Black	Brown	Green
Surface morphology	Flat, without algal filament	Bit rough, with algal filament	Very rough, rosulate of thallus	Rough, crustose of thallus	Bit rough, blanket of moss
Dominant species	*Microcoleus vaginatus*	*Scytonema javanicum*	*Nostoc* sp.	*Apatococcus* sp.	*Byrum argenteum*
Coverage (%, dry/moist)	>95/100	>90/100	>60/90	>75/85	>90/100
Thickness (mm)	4.65 ± 0.52^a^	6.58 ± 0.54^b^	9.70 ± 0.86^c^	11.01 ± 0.78^d^	13.63 ± 0.74^e^
Sand (%)	94.24 ± 0.18^a^	89.91 ± 0.39^b^	63.33 ± 1.35^c^	64.07 ± 0.23^c^	58.19 ± 0.07^d^
Silt and clay (%)	5.76 ± 0.16^a^	10.09 ± 0.45^b^	36.67 ± 1.34^c^	35.93 ± 0.18^c^	41.81 ± 0.08^d^
Porosity (%)	32.37 ± 0.13^a^	40.88 ± 1.48^b^	53.07 ± 1.61^c^	54.62 ± 0.75^c^	57.75 ± 0.47^d^
OM (mg cm^−2^)	6.63 ± 0.34^a^	12.92 ± 0.69^b^	41.42 ± 2.80^c^	46.75 ± 1.92^d^	80.52 ± 3.93^e^
Chl a (μg cm^−2^)	8.37 ± 0.57^a^	9.67 ± 0.98^a^	29.26 ± 2.05^b^	33.85 ± 3.81^bc^	37.60 ± 3.67^c^
Total Biomass (μmol CO_2_ h^−1^cm^−2^)	1.41 ± 0.16^a^	1.88 ± 0.19^b^	2.75 ± 0.09^c^	3.30 ± 0.25^d^	4.91 ± 0.09^e^

The different superscript letters represent that the differences are significant (P < 0.05). EPS = Exopolysaccharides, OM = Organic matter.

### Simulated experiments

The activities of biocrusts were reactivated three days before experiments. Samples in 5 cm-diameter lids were rehydrated with sterile distilled water and then put into a microclimate with a light intensity of 100 μmol m^−2^ s^−1^ and ambient temperature and CO_2_ concentration, until the weight of biocrusts dropped to the desiccation level. Each type of biocrusts was tested with 3~5 replications. The whole simulated experiments were completed within 3 months. During the experiments, SW and equivalent NRW in mm were separately added to biocrusts. The NRW amount was based on the actual values measured in the field in autumn, and it was added uniformly to the surface of biocrusts with a small atomizer. Next, the CO_2_ exchange rate of biocrusts at various temperatures (−2.5 °C, 0 °C, 2.5 °C, 5 °C, 7.5 °C, and 10 °C) and light intensities (dark to 400 μmol m^−2^ s^−1^, based on the field light irradiation from night to early morning) was measured using a soil carbon release rate determination device (Yaxinliyi Sci Technol Co. Ltd of China, resolution 0.01 μmol CO_2_ m^−2^ s^−1^). CO_2_ concentration was the ambient value. During measurements, biocrusts samples were put into a transparent chamber of 0.16 L that was connected to a measurement system. The temperature in the chamber was regulated with a water circulation system, light intensity was controlled with an external light source and light meter (Hansatech Instruments Ltd., UK), and the gas flow rate was controlled at 0.6 L min^−1^ by an internal mini air pump and flowmeter. The measurements were automatically converted into μmol CO_2_ m^−2^ s^−1^ by the host system. The carbon exchange rate in darkness represented the dark respiration of the biocrusts. The gross photosynthesis rate (Pg) was calculated from the CO_2_ exchange rate measured under darkness and light at a specific temperature.

### Field mesocosm experiments

Mesocosm experiments were conducted in the Field Experiment Station of the Hobq Desert (40°21′N, 109°51′E) in September, 2014 and 2015. Newly collected samples were placed in open areas between sand dunes as soon as possible after harvesting, more than 2 m away from vascular plants. The bottoms of biocrust samples were adjoined the sands below and the upper surfaces of biocrusts were in the same horizontal plane with soil surface, just like under natural conditions. Each sample area was big enough to ensure real thermal conditions and temperature changes between night and day. Care was taken to maintain the natural thickness and completeness of samples during collection, transportation, and placement. The measurement interval was 1 h from 18:00 to 5:00 a.m., and was 0.5 h after that. At least 30 replicates were set up for each type of biocrust samples. Properties of biocrusts including thickness, coverage, roughness, and other feature, were kept as consistent as possible. All samples were measured at different time points until carbon exchange was no longer detected after sunrise. Samples were also set to monitor the real-time NRW amount at different time points. Microclimate parameters (including temperature, light intensity, and air relative humidity) were synchronously recorded by climate observatory and light meter.

### Data analysis

Variances of CO_2_ exchange rate response to the combinations of different temperature and light intensity were analyzed by one-way ANOVA at the 95% confidence degree. The relationships between CO_2_ exchange rate and temperature and light intensity were analyzed by multiple regression analysis. All above analyses were performed by using SPSS 18.0 software. The influences of light intensity, temperature and real-time NRW amount on the carbon exchange rate of biocrusts were analyzed using structural equation models. Data modeling was conducted on Amos 17.0 software.
